# Infrastructural Limitations in Establishing Neurosurgical Specialty Services in Liberia

**DOI:** 10.7759/cureus.29373

**Published:** 2022-09-20

**Authors:** Ira Bowen, Harjyot Toor, Bailey Zampella, Alvin Doe, Christopher King, Dan E Miulli

**Affiliations:** 1 Neurosurgery, Riverside University Health System Medical Center, Moreno Valley, USA; 2 Neurological Surgery, John F. Kennedy Memorial Hospital, Monrovia, LBR; 3 Neurosurgery, Aurora Medical Center, Summit, USA; 4 Neurosurgery, Arrowhead Regional Medical Center, Colton, USA

**Keywords:** international neurosurgery, neurosurgery in developing countries, infrastructure, liberia, africa

## Abstract

Introduction

Liberia recently employed the first neurosurgeon in the country’s history. In a country with a population of 4.7 million people and staggering rates of cranial and spine trauma, as well as hydrocephalus and neural tube defects, neurosurgery is considered a luxury. Our study documents the experience of a team of neurosurgeons, critical care nurses, scrub technicians, nurses, and biomedical engineers who carried out a series of neurosurgical clinics and complex brain and spine surgeries in Liberia. Specifically, we aim to highlight some of the larger obstacles, beyond staff and equipment, facing the development of a neurosurgical or any other specialty practice in Liberia.

Methods

Our institutions, in collaboration with the Korle-Bu Neuroscience Foundation, spent 10 days in Liberia, based in Tappita, and performed 18 surgeries in addition to seeing several hundred clinic patients. This is a retrospective review of the cases performed along with outcomes to investigate obstacles in providing neurosurgical services in the country.

Results

Before arriving in Liberia, we evaluated, planned, and supplied staff and materials for treating complex neurosurgical patients. Sixteen patients underwent 18 surgeries at a hospital in Tappita, Liberia, in November 2018. Their ages ranged from 1 month to 72 years (average 20 years). Five patients (28%) were female. Ten patients (56%) were under the age of 18. Surgeries included ventriculoperitoneal shunting (VP-shunt), lumbar myelomeningocele repair, encephalocele repair, laminectomy, and a craniotomy for tumor resection. Ten patients (55%) underwent VP-shunting. Two patients (11%) had a craniotomy for tumor resection. Three patients (17%) had laminectomy for lumbar stenosis. Two patients (11%) had repair of lumbar myelomeningocele.

Conclusion

After an aggressive and in-depth approach to planning, conducting, and supplying complex neurosurgical procedures in Liberia, the greatest limiting factor to successful outcomes lie in real-time is access to health care, which is largely limited by overall infrastructure. Our study documents the experience of a team of neurosurgeons, critical care nurses, scrub technicians, nurses, and biomedical engineers who carried out a series of neurosurgical clinics and complex brain and spine surgeries in Liberia. Specifically, we aim to highlight some of the larger obstacles, beyond staff and equipment, facing the development of a neurosurgical or any other specialty procedural practice in the country of Liberia. Most notably, we focus on infrastructure factors, including power, roads, water, education, and overall health care.

## Introduction

Liberia recently employed the first neurosurgeon in the country’s history. In a country with a population of 4.7 million people [1] and staggering rates of cranial and spine trauma, as well as hydrocephalus and neural tube defects, neurosurgery is considered a luxury [2,3]. A countrywide survey in Liberia showed a shortage of essential surgical procedures [4]. The national density of surgical providers is only 6.7/100,000, the density of specialist providers is 1.6/100,000, and the density of neurosurgeons is 0.02/100,000 [5,6]. Currently, patients in need of neurosurgical care are required to travel to other countries, often as far as Ghana or India. Given the fact that for the average Liberian family, even simple medications such as antibiotics pose a significant financial burden, most patients are simply forced to go without care and let pathologies take their natural course. The natural course of neurosurgical illness is typically devastating. The prospect of developing a neurosurgical center within Liberia has been met with much excitement from both the civilians and the government. Much of this development has been aided by the Korle-Bu Neuroscience Foundation, which has facilitated recent trips to Liberia with American and Canadian neurosurgeons to perform surgeries, train staff, and establish basic neurosurgical care. Unfortunately, while these efforts have been largely successful in introducing neurosurgery to local hospitals, a great divide remains in the overall infrastructure that is required to maintain and adequately perform complex surgeries in the country. Our study documents the experience of a team of neurosurgeons, critical care nurses, scrub technicians, nurses, and biomedical engineers who carried out a series of neurosurgical clinics and complex brain and spine surgeries in Liberia. Specifically, we aim to highlight some of the larger obstacles, beyond staff and equipment, facing the development of a neurosurgical or any other specialty practice in the country of Liberia. A self-sustaining healthcare system and infrastructure are necessary to create a foundation upon which surgical practices can be created. This applies to any area where primary care, education, and basic needs are lacking, such as in rural areas in the United States. Here, we focus on infrastructure factors, including power, roads, water, education, and overall health care. Additionally, we evaluate the feasibility and challenges associated with establishing a neurosurgical center in rural Tappita versus within the country’s capital and population center of Monrovia. 

Background

The country of Liberia has struggled with multiple recent civil wars, including the First Liberian Civil war between 1989 and 1997 and more recently, a coup in 2003. The initial civil war resulted in the death of 250,000 Liberians, over 5% of the population. These internal conflicts have halted development and left the country as one of the most underdeveloped countries per GDP in Africa, according to the 2018 World Bank forecast report for Liberia. Despite the continued political and social unrest, with the aid of China, Liberia was able to open a hospital in 2011 in Tappita, which was designed to serve as a specialist referral hospital with capabilities of advanced surgery. The hospital, in its infancy, dealt with the Ebola epidemic in 2014, and an already tenuous developing infrastructure was further handicapped [1]. Regardless, the people of Liberia have persevered and continued to establish a growing medical practice in the area of Tappita. Currently, the Liberian government has defined this hospital as the specialty referral center for the country and emphasized its development for the most complex patient cases.

Unfortunately, many obstacles continue to hinder the capabilities, outcomes, and growth of the hospital in Tappita. The most immediately obvious obstacle is access to care which includes providers, roads, and transportation. The successful development and sustainability of neurosurgery, or any procedural-based specialty, service is like the successful development and sustainability of a country. This can be seen in the development of neurosurgical programs in other developing countries in Africa [7,8]. In both, the requirements of power, roads, water, and education are paramount. Power, whether in a developing country or developing a neurosurgical program, depends upon the ability to influence, whether through people, environment, energy, or equipment, the desired outcome. Building a city and building a complex specialty-specific program in the hospital requires roads; the ability to get from one point to another, such as travel, and also the organization’s desires, protocols, plans, and procedures to achieve the outcome. Water not only aids in the delivery of sustainable nourishment and removal of waste but also is an intricate service dependent upon providing accurate amounts of fluids and removing waste products to achieve the patient's best outcome. Education is the ability to teach others, gather data, investigate, fix, maintain, and improve the quality of a city and premier neurosurgery or other procedural-based services [9]. Following 14 years of civil war, approximately 80% of clinics throughout the country were closed secondary to looting, destruction, and flight of healthcare personnel [10]. This was another barrier to successfully developing a neurosurgical program.

While Monrovia is home to 90% of Liberia’s population, the city’s main hospital is severely limited in resources. At the time of the 2018 trip, Monrovia’s limitations included a lack of magnetic resonance imaging (MRI) and computerized tomography (CT) scanners, no functional ventilators, as well as grossly underequipped operating rooms. As such, patients requiring a higher level of neurosurgical or neurological care are routinely transported to the hospital in Tappita, 220 miles away, where more advanced care is available. However, to make this journey, families have two difficult options. The best option involves a short flight for the price of $100 (US dollars). For the average Liberian patient, this falls into the “luxury” category and is entirely unaffordable. The second and more frequently employed option is traveling by vehicle. Despite the relatively short distance, the difficulty of this journey cannot be underestimated. While the first few hours after leaving Monrovia are on a paved road, the true journey begins around Ganta, Liberia, when the road becomes a narrow, unpaved mud path complete with multiple river crossings and obstacles. During the dry season, this dangerous, tumultuous commute can take 6-8 hours, but during much of the year, with heavy rains and muddy conditions, the drive takes 10-12 hours at best. During the day-long drive, usually in the back of a necessary four-wheel drive vehicle, the passengers are subjected to nearly constant potholes, crevasses, and at least a half dozen crossings where they are required to be towed or pulled by winch through river crossings. Patients leaving the hospital in Monrovia are transported via “ambulance”, which is simply a pickup truck with a covered bed, a single cot, and a siren. In reports as recently as weeks before our group’s 2018 trip, patients with traumatic spinal cord injuries were sent from Monrovia to Tappita and unfortunately expired during the drive. Approximately 70% of patients seen by our team at the neurosurgical clinic in Tappita had traveled via this road, despite their neurological injuries. 

Setting aside the obvious difficulties associated with transporting patients to Tappita’s hospital, a host of infrastructural limitations continue to impede the process of medical treatment and healthcare growth. Electrical power to the hospital is provided by multiple diesel generators. These generators require switching the power source, on average, twice daily. This process means power is rarely available between 7 and 8 am, and an additional 2-10 minutes power outage daily around 5 pm while the generators are switching over. While the intermittent power outages provided slight obstacles in the operating room or clinic, they guarantee that the MRI machine will never be functional as it requires a consistent power source. As a result, Liberia’s only MRI remains disassembled and boxed in storage in Tappita. 

While adequate water supply does exist in the region, potable water was in short supply. Fluids of any sort for patients were predominately supplied by family members and not readily available through the hospital pharmacy. Additionally, patients largely depend on family members for nutrition, hygiene, and bathing during their hospital stay, as this was not in the daily routine of the nursing staff.

In the rare event that all the above obstacles were overcome, the patients were evaluated, medically cleared for surgery, admitted to the hospital, and successfully able to undergo various complex neurosurgical procedures. While the ability to perform complex surgeries had many organizational limitations that were able to be overcome through planning, personnel, and education and is discussed in detail in additional reports, the delivery of perioperative care remained one of the biggest challenges. The Liberian people have incredible drive, resilience, and many educated nurses, but overall education and exposure to critically ill patients in a postoperative setting are severely limited. Similarly, the lack of optimization of this exceptional hospital facility limits the local providers’ ability to flawlessly care for patients. The established “Neurosurgical Intensive Care Unit (ICU)” consisted of an open-air room where multiple patients were placed in beds with mosquito netting and a limited amount of pulse oximetry machines which are utilized on a first-come, first-serve basis. Physicians and nurses in the “ICU” had access to a handful of medications, including ceftriaxone and acetaminophen, but very little beyond that. For example, the lack of access to basic anti-hypertensive medications in postoperative neurosurgical patients provided an obvious barrier in management, if it were not for supplies brought from the US and Canada. Further, the concept of hourly neurological checks and checking vitals were not common practice. In our experience, the nursing staff was incredibly intelligent and open to hands-on education and adapting to new practices but had simply never been given the opportunity. US-based critical care neuro-ICU nurses who were part of our team spent many hours educating nurses and found them to be very interested in adopting new practices, but again, were met with limited resources and a lack of general background knowledge. Education was a notable shortfall, with very few nursing staff aware of basic neurosurgical criteria such as the Glasgow Coma Scale (GCS), neurological exam, and the necessity of frequent vitals. We understood before our journey that the nurses and staff were not exposed to neurological diseases in the past, and therefore part of our primary mission was to train them.

Upon discharge from the hospital additional obstacles arose for continued patient care. As mentioned before, the most obvious and physical obstacle that epitomizes the lack of infrastructure can be seen in the roads. The difficulties of travel on these roads include both the unpredictable and dangerous conditions, as well the sheer distance often required. For instance, while most patients live in the city of Monrovia, the majority of neurosurgical resources and the local neurosurgeon are located in the town of Tappita, many hours away. While the local neurosurgeon does make the trip from Tappita to Monrovia every couple of weeks to see patients at the hospital in Monrovia, this is obviously not a feasible option in the setting of a clinically declining neurosurgical patient located either in Monrovia, Tappita, or other parts of the country. Further challenges arise when families are often required to pay for each individual follow-up appointment out of pocket. In a region of extreme poverty, many patients are quickly lost to follow-up simply secondary to financial difficulty. Lastly, an accessible system of online or telephone prescriptions is not established. Therefore, patients requiring medication must travel the distance and pay to see the physician, where they then must purchase the medication from the hospital or pharmacy. As a result, there are frequent delays in delivery of antibiotics, and often patients do not receive the necessary medication at all. Follow-up care is further limited as primary care physicians are not comfortable seeing patients of specialists even in routine postoperative settings. 

## Materials and methods

**Figure 1:**
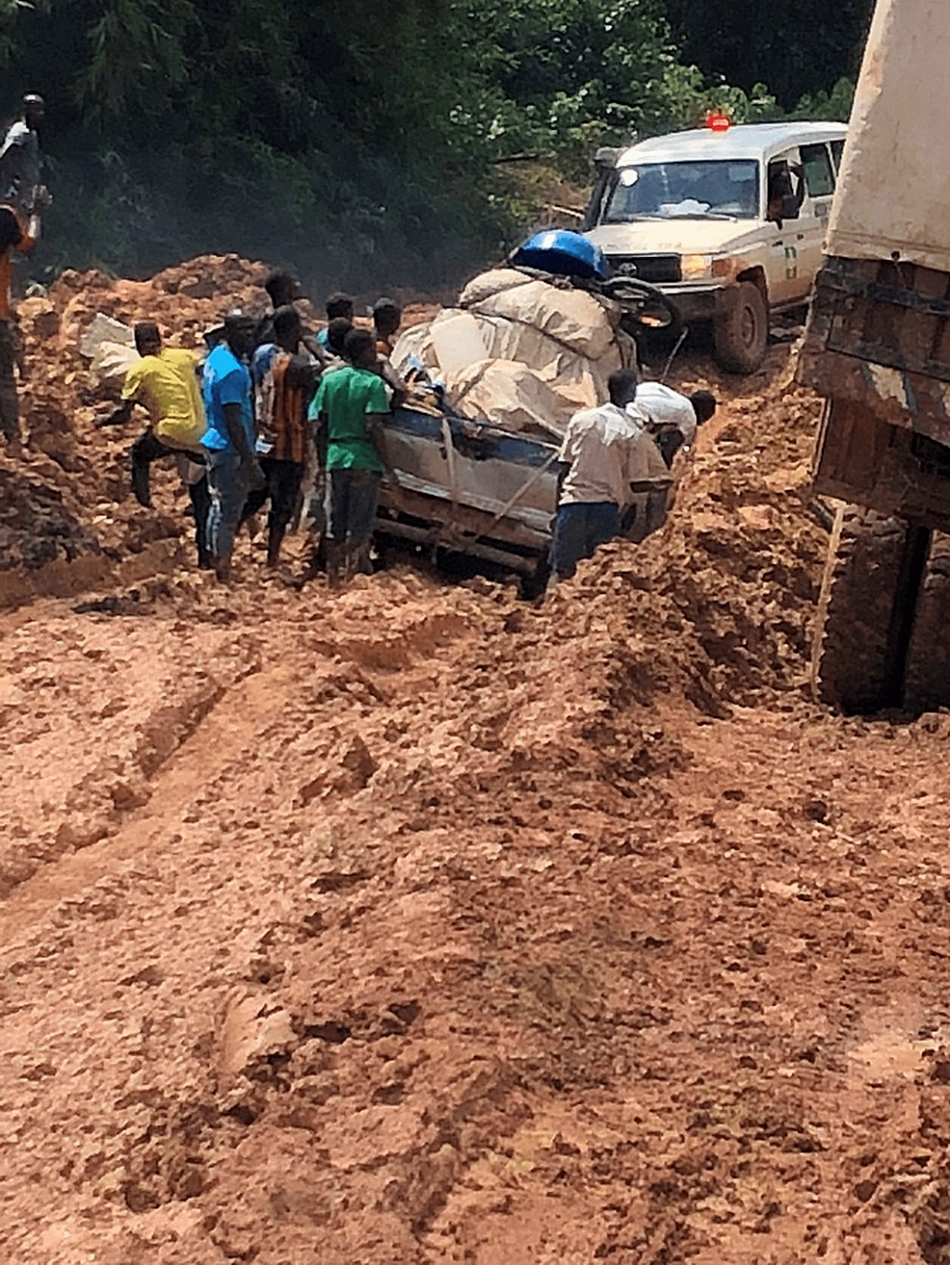
Photograph of only road entering Tappita.

This study was approved by the Arrowhead Regional Medical Center Institutional Review Board (Protocol #21-40) and in conjunction with the only neurosurgeon in Liberia. Our institution, in collaboration with Korle-Bu Neuroscience Foundation, spent 10 days in Liberia, based at a hospital in Tappita, and performed 18 surgeries in addition to seeing several hundred clinic patients. Patient demographics, preoperative physical assessment, surgery performed, postoperative physical assessment, postoperative care, and 30-day morbidity and mortality were recorded. During their perioperative hospital stay, patients were evaluated based on vitals, GCS, neurological exams, labs, and imaging. These evaluations were limited by resources. For instance, at the time of this trip, an X-ray and CT was available, but MRI was not. Myelography was not available due to the low availability of contrast. Basic labs, including complete blood count (CBC) and bleeding time were accessible but used sparingly as the cost burden fell upon patients and their families. Basic metabolic profiles (BMP) were not available because the laboratory analyzer had failed multiple times, along with countless other devices. Fortunately, our bioengineers were able to repair many of the instruments. Antibiotics were limited to ceftriaxone and gentamycin and supplemented by our enduring supply. Constant bedside monitoring involved pulse oximetry, which also provided heart rate; however, no continuous monitoring of heart rhythm, temperature, or blood pressure was available. Intake and output were recorded by nursing staff but were based on the need for Foley drainage and not assessed hourly. Nutrition and hygiene were largely provided by the patient's family members. Follow-up healthcare consisted of visits in either Tappita or Monrovia with the only local neurosurgeon and recurring monthly clinics between the US and Liberian neurosurgical teams via video conference. The main method of transportation was one road in and out of Tappita, as discussed earlier (Figure 1).

## Results

Prior to arriving in Liberia, we evaluated, planned, and supplied staff and materials for treating complex neurosurgical patients. Sixteen patients underwent 18 surgeries at the hospital in Tappita, Liberia, in November 2018. Their ages ranged from 1 month to 72 years (average 20 years). Five patients (28%) were female. Ten patients (56%) were under the age of 18. Surgeries included ventriculoperitoneal shunting (VP-shunt), lumbar myelomeningocele repair, encephalocele repair, laminectomy, and craniotomy for tumor resection. Ten patients (55%) underwent VP-shunting. Two patients (11%) had craniotomy for tumor resection. Three patients (17%) had laminectomy for lumbar stenosis. Two patients (11%) had repair of lumbar myelomeningocele. Two patients (11%) had repair of encephalocele. Of the two patients with encephalocele, one with a nasal encephalocele underwent concomitant bi-frontal craniotomy for repair of bony frontal defect and dural closure. All patients were placed on a 24-hour course of ceftriaxone postoperatively. The course of antibiotics was extended as needed for patients with shunts at the discretion of local neurosurgery team during perioperative follow-up. Nurses were instructed to record hourly neurological exams and vitals. Adverse events are outlined in Table 1.

**Table 1:** Adverse events. mo: Months old, mo: Years old, VPS: Ventriculoperitoneal shunt, POD: Postoperative day

Age	Diagnosis	Surgery	Adverse event	Timing (post op)	Cause	Disposition
24mo	Hydrocephalus, meningocele	VPS and meningocele repair	Acute respiratory failure	Immediately post-op	Inadequate ventilation intra-op	Expired
1mo	Encephalocele, hydrocephalus	VPS, encephalocele repair	Wound drainage	POD7	Superficial infection	Resolved with antibiotics
6yo	Hydrocephalus	VPS	Shunt infection	POD23	Shunt infection	Expired
48yo	Craniopharyngioma	Bifrontal craniotomy for tumor resection	Acute respiratory failure	POD23	Mucus plug vs pulmonary embolism	Care withdrawn per family's request, expired
8yo	Encephalocele	Encephalocele repair	Subgaleal fludi collection	POD30	Proximal shunt failure	Shunt revised, recovered to baseline

Each of the three patients undergoing laminectomy for lumbar stenosis with radiculopathy were evaluated immediately postoperatively, prior to discharge, and at the one-month mark. All three patients endorsed resolution of preoperative radicular pain. One patient continued to have preoperative knee pain due to known chronic degeneration of the knee requiring surgery. One patient returned to the hospital for evaluation of the wound. The patient was concerned with suspected serous drainage from the wound. Symptoms were inconsistent with infection. The patient was observed overnight in the hospital, and no drainage was noted from the wound. 

Each patient who developed any postoperative issue was best served by being evaluated in the hospital setting, as outpatient care for specialty patients is not available. Almost all patients experienced unexpected degree of difficulty in obtaining appropriate perioperative care, with the most common obstacles being travel due to weather, access to medication, and access or affordability of clinic visits (Table 2). 

**Table 2:** Obstacles encountered for perioperative care.

Obstacle	Number (Percentage)
Travel >5 hours	12 (75%)
Unable to obtain recommended medication	4 (25%)
Unable to make in person follow-up within 30 days – secondary to cost or distance	7 (44%)
Unable to afford recommended post-operative treatment	2 (12.5%)

## Discussion

The impact of establishing neurosurgical care in a developing county comes with a variety of obstacles beyond simply providing care. At first glance, even the most educated benefactor may jump to the conclusion that the answer lies in providing surgical tools, medication, and physical resources. However, following an immersive initiative by an American and Canadian neurosurgical team, it became apparent that the limitations of successful neurosurgery or any surgical specialty within the county of Liberia lies in healthcare infrastructure. While our team of neurosurgeons, surgical technicians and nurses, ICU nurses, and biomedical engineers were able to bring equipment and supplies to carry out some of the most complex skull base and spine surgeries as well as routine cases, little is achieved if the patients are not able to receive basic perioperative, postoperative, as well as primary care. In order to facilitate, evaluate, and understand the health care circumstances, we held several meetings over the course of months before we visited Liberia to ensure that proper perioperative care would be available to the patients. We also planned for frequent subsequent visits. However, as seen with the patients we operated on, there are barriers to medical care beyond having these issues addressed beforehand. After all of the surgeries and returning to the US, we continued to hold meetings to review what patients needed in terms of post-operative and primary care, and what would be needed to provide for patients in the future. Patients often are unable to access follow-up care due to inherent financial limitations, lack of transportation, time off work, and severe weather conditions. Illiteracy and poor understanding of the necessity of follow-up care, despite attempts to educate patients, can lead to loss of follow-up. In addition, other external factors limiting care include shortages of medication related to supply, misplacement, theft, transportation, and financial limitations. Further, despite the availability of advanced operating rooms and neurosurgeons, nothing is gained if the patient cannot physically endure the journey to the hospital. Our neurosurgery program remains intimately involved with the country of Liberia and their budding neurosurgery program and planned frequent medical missions to address issues identified as well as teach and educate physicians, nurses, technicians, managers, and healthcare administrators.

In the days and weeks following surgery, the most apparent barrier to successful neurosurgical care in Liberia became, as it is in many developed countries, access to medical care. The majority of patients who underwent surgery in Tapitta, 11 (70%), lived in Monrovia, the capital of Liberia. Follow-up care, if any, was scheduled in Tappita, a 10-12-hour drive over difficult and impassable roads. While the hospital in Tappita did have more advanced facilities and healthcare, the risk of patient transportation, the almost certain loss of follow-up, and the divide between patient and neurosurgeon, or any healthcare provider, nearly guarantees an unacceptable postoperative course. As stated earlier, although we tried to ensure that follow-up care would be obtainable and mandatory for patients who had undergone surgery, external factors caused patients to be lost to follow-up. We were able to return in 2019, at which time our original plan was to help establish the surgical center in Tappita. However, given the lack of infrastructure, particularly in healthcare administration, as well as the long distance between the main population in Monrovia in need of healthcare and the hospital in Tappita, we instead made a trip to Monrovia and provided neurosurgical care there for one week. Other US and Canadian teams planned to return frequently to Liberia after that time, but the coronavirus pandemic prevented a trip in 2020. The difficulties that patients had without the planned follow-up were revealed during the pandemic given our inability to visit again.

Following our experience in Liberia, we would recommend that neurosurgical or any procedural-based specialty care become focused in a single hospital in a larger metropolitan area with consistent access to real-time care during the entire peri- and postoperative period. While the capabilities of the hospital in Tappita initially exceeded those of the hospital in Monrovia, future development in Tappita's infrastructure will likely yield minimal results without drastically improved patient healthcare access. There is a need for reliable supply chains and education of personnel for obtaining, managing, and utilizing supplies. Additionally, medical literacy must be improved in patients, their families, and the communities that support them. The importance of perioperative care cannot be stressed enough in order to improve outcomes for the life-saving surgeries that we aim to provide.

A detailed review of the source of each adverse event came to a similar conclusion, which was the lack of access to immediate patient care. In each case, this lack of access to care was secondary to obstacles not generally encountered in the US. In Liberia, the obstacles to successful neurosurgical care are the lack of access to healthcare in the forms of cost, distribution of antibiotics and other medication, power supplies for equipment, more reliable equipment, distance and travel modalities to a hospital, sanitation, availability of primary physician care, and education about advanced specialty care. Each of these complications, and ultimately deaths, would have been avoidable in the United States secondary to easier access to basic health care, along with increased medical literacy and the ability to continuously follow up with patients. There is a need for basic primary care in addition to peri- and post-operative care. Performing neurosurgery or any specialty procedure with minimal equipment is a challenge to the surgeon’s capabilities and foresight; however, taking care of neurosurgical patients postoperatively requires access to a team of both highly trained and highly equipped personnel, along with buy-in from patients, their families, hospital management, and their communities [2,3].

## Conclusions

After an aggressive and in-depth approach to planning, conducting, and supplying complex neurosurgical procedures in Liberia, we have found the greatest limiting factor to successful outcomes lie in real-time access to health care. While the hospital in Tappita clearly provides the highest level of available care, this is of little use to most patients living outside of that hospital’s catchment area. Monrovia is the nation’s capital and population center, and despite the minimal short comings of its metropolitan hospital, patients would have a far greater chance of overcoming disease if they could always have access to any type of healthcare. It is unrealistic to assume that any patient with a neurosurgical disease can both afford and tolerate the exhausting 10-12-hour tumultuous journey to Tappita. Further, with 90% of the patient population focused in a single city, it only makes sense to focus advanced resources there until the geographically inaccessible region is made accessible. 

Despite the efforts of outside forces, and as highlighted by an unexpected barrier for the US and Canada teams to return to Liberia due to the coronavirus pandemic, the true success will fall with the ability of local medical and political forces to provide access to adequate care. Without the proper infrastructure, including healthcare administration, any medical mission will not be successful. Similarly, the end goal is not to develop a program reliant on outsiders but to foster a relationship that will grow neurosurgery and other specialties in Liberia through basic principles, organization, education, and access to care. After ample exploration and in-depth practice review, we would ultimately recommend that current neurosurgical efforts be supplemented by consistent and reliable access to care. During the interim, neurosurgery postoperative care must be provided continuously, 24 hours per day, in both Tappita and Monrovia. Our mission is to influence a national change focusing from a distant center for advanced specialty care through organization and access, to deliver sustainable health care and an environment of education, investigation, and quality improvement for all. This first trip to Liberia was necessary to evaluate and understand patient care firsthand. It was different from background information obtained before the journey. However, this allowed us to plan and improve subsequent trips.
